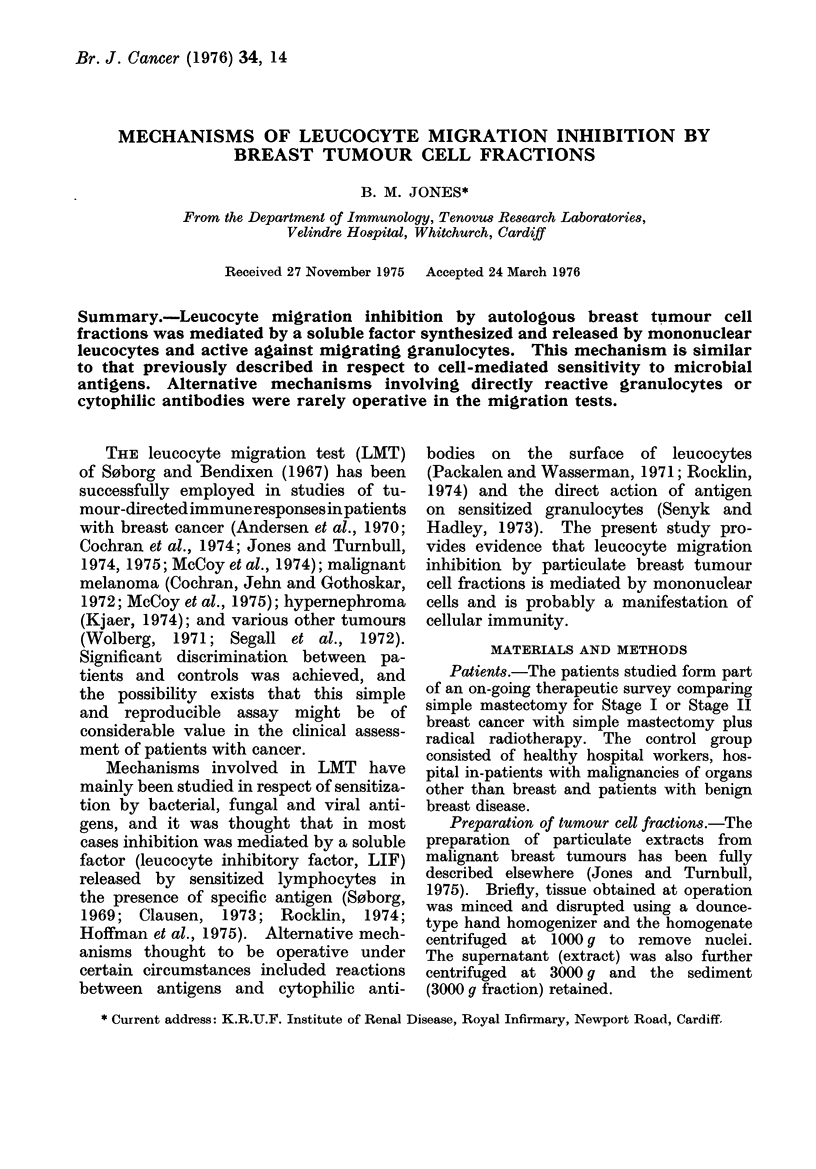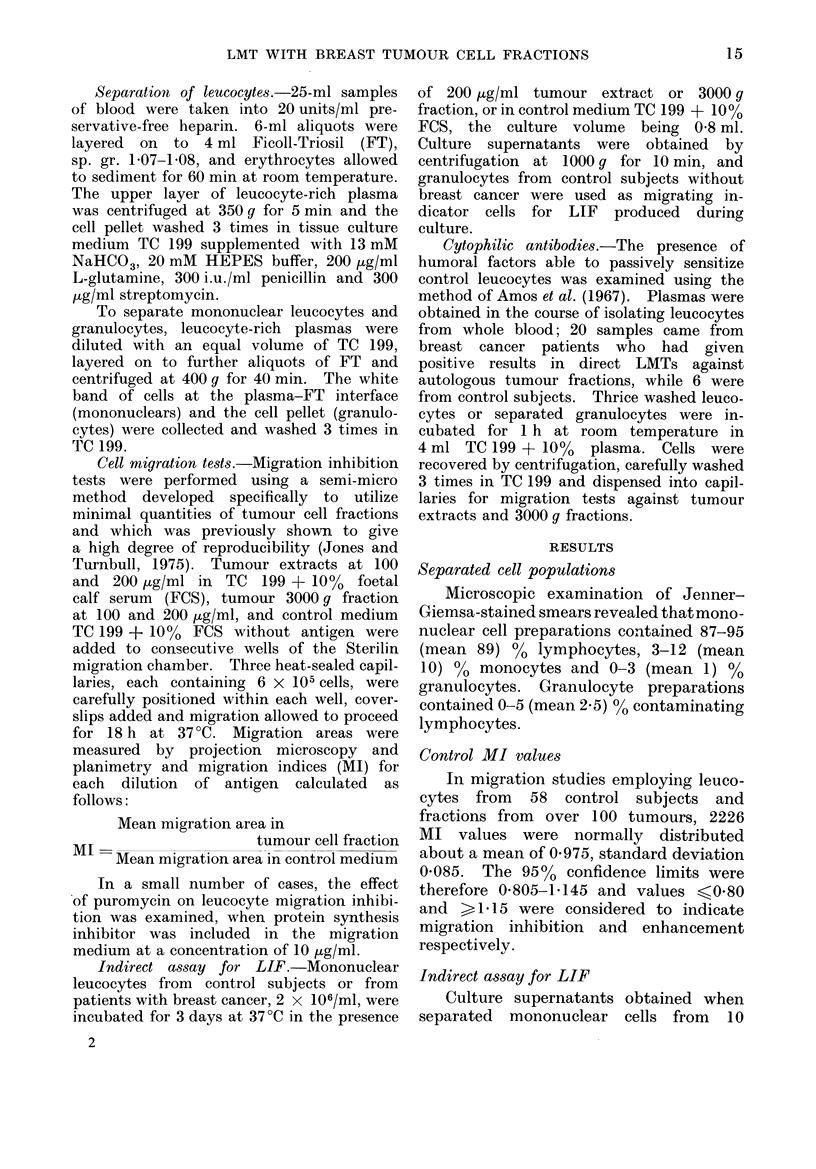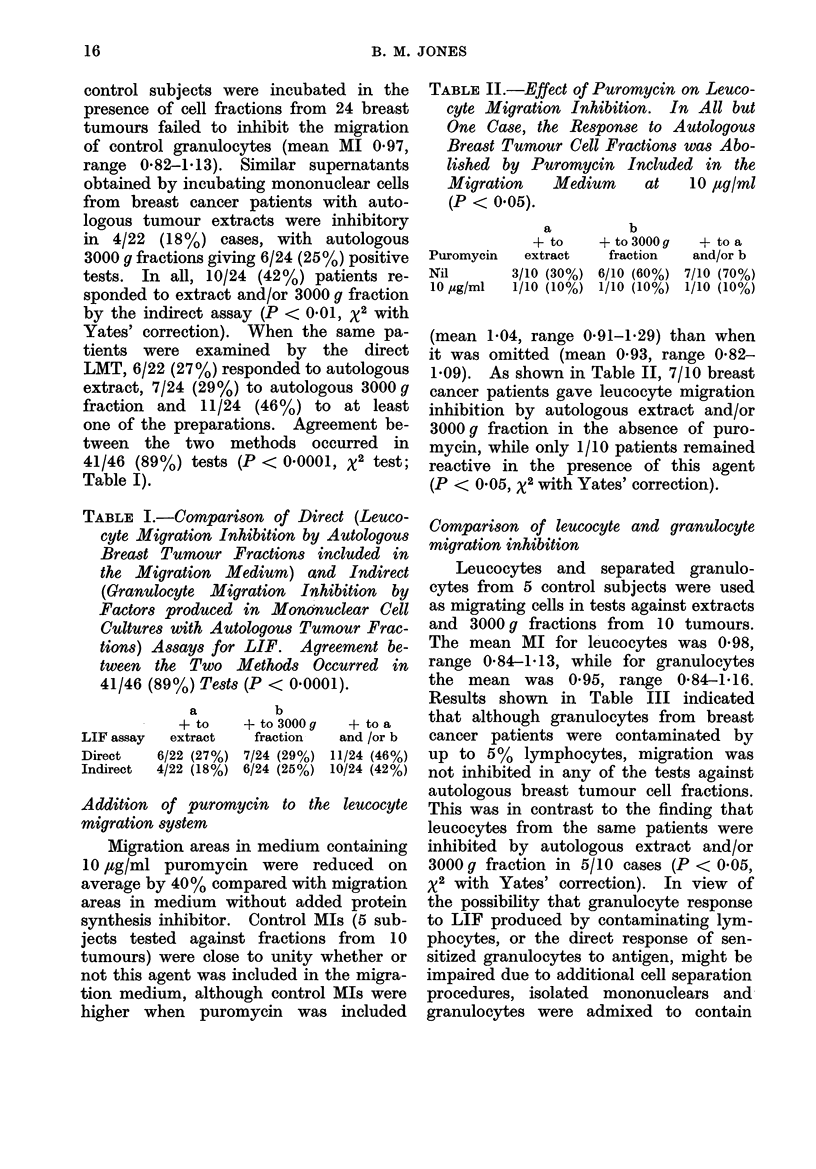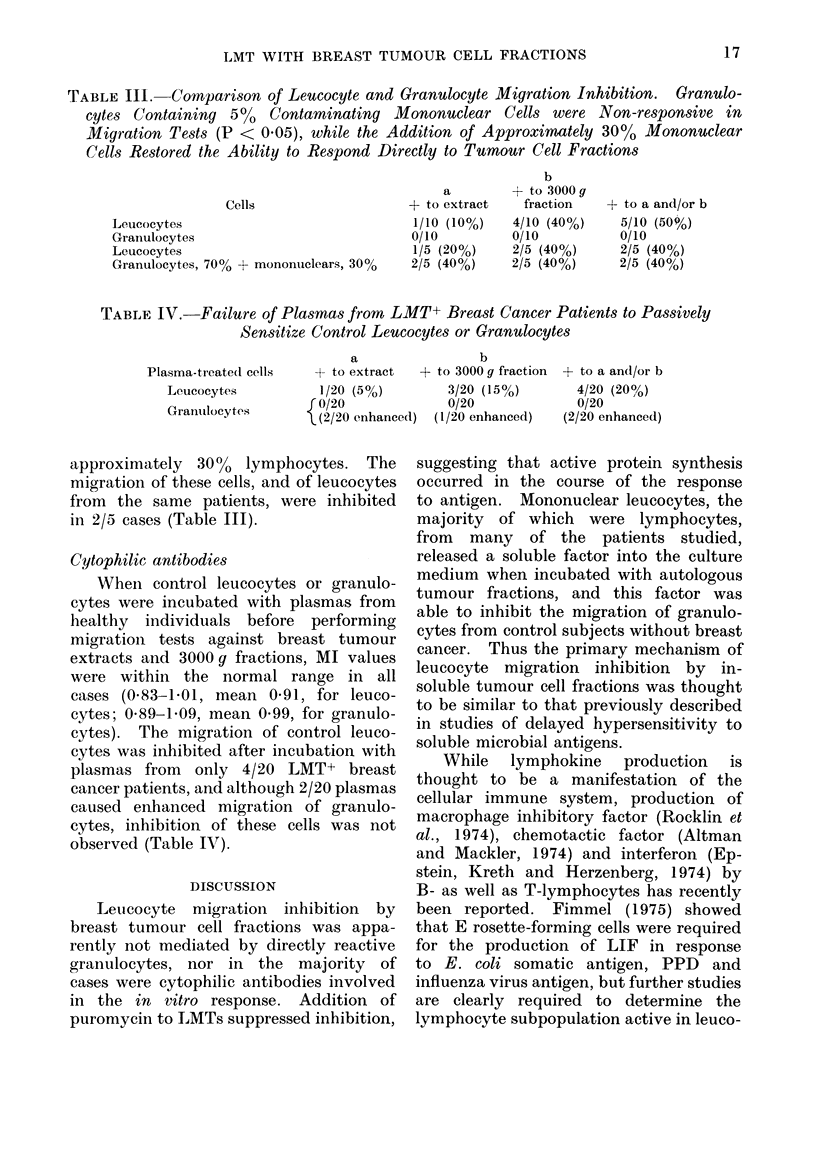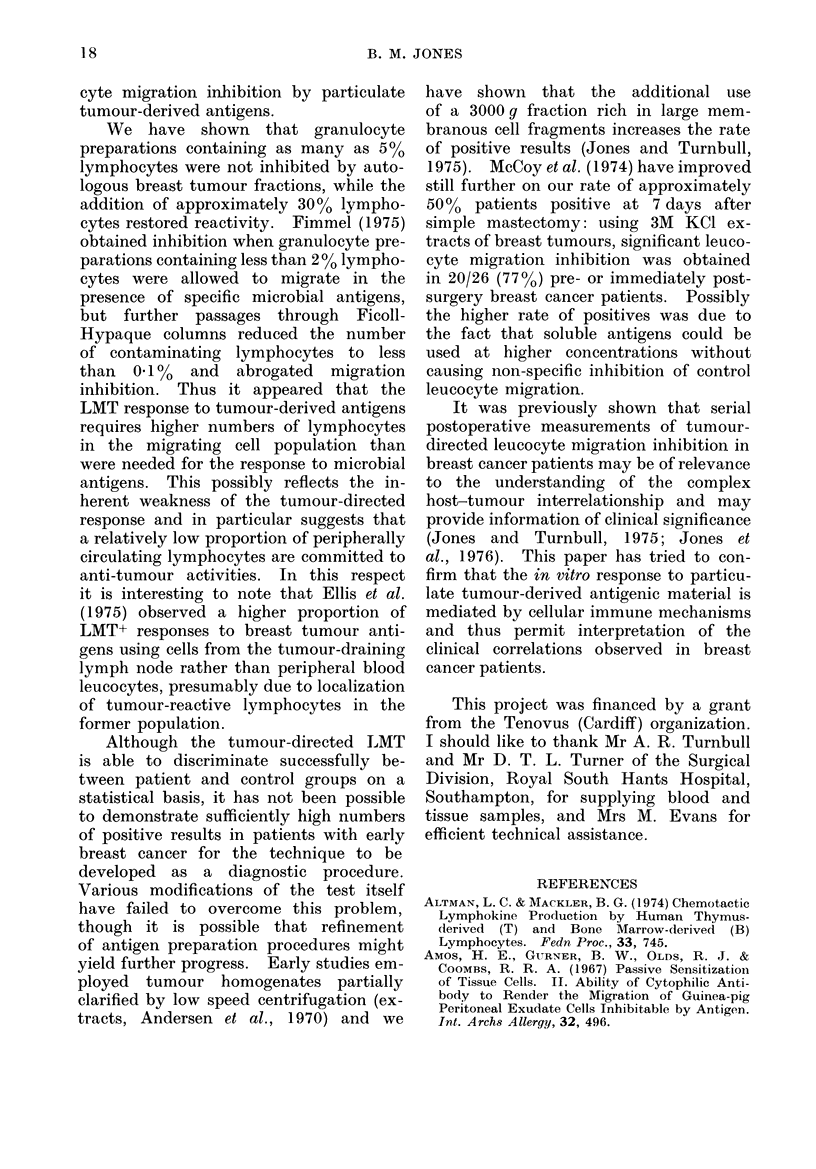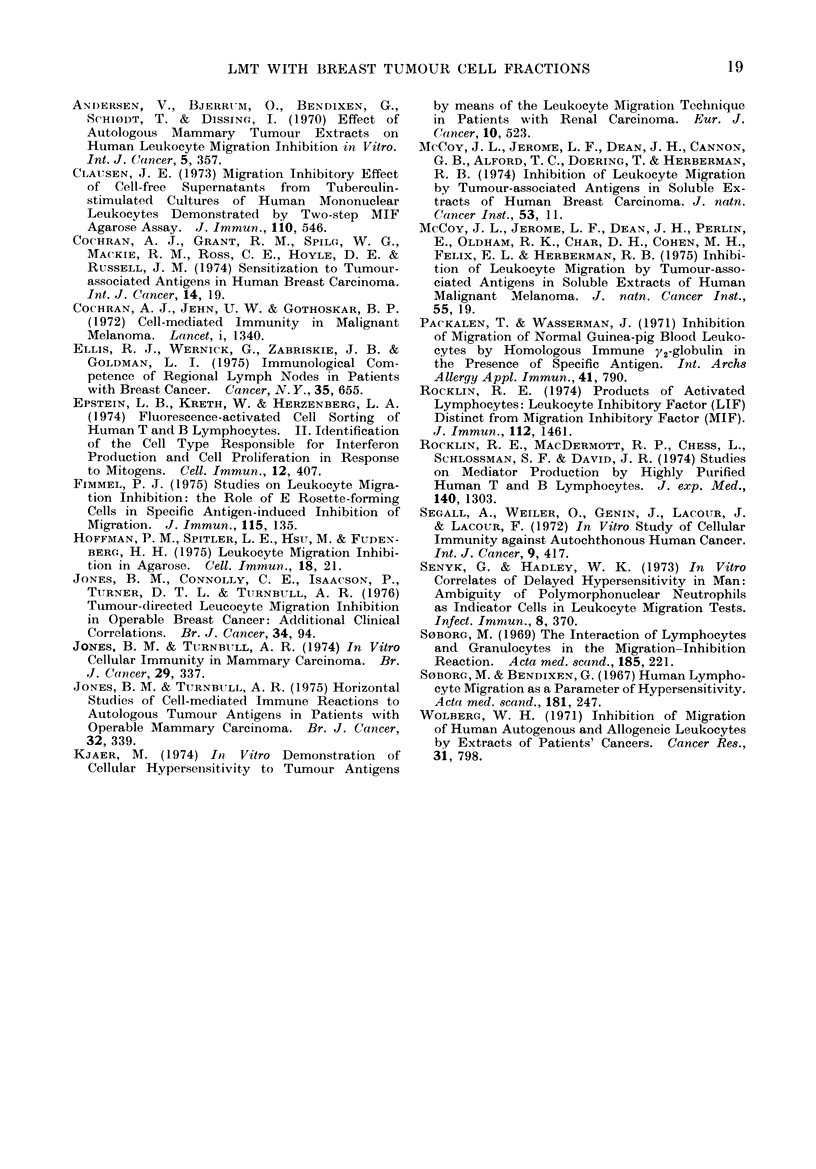# Mechanisms of leucocyte migration inhibition by breast tumour cell fractions.

**DOI:** 10.1038/bjc.1976.115

**Published:** 1976-07

**Authors:** B. M. Jones

## Abstract

Leucocyte migration inhibition by autologous breast tumour cell fractions was mediated by a soluble factor synthesized and released by mononuclear leucocytes and active against migrating granulocytes. This mechanism is similar to that previously described in respect to cell-mediated sensitivity to microbial antigens. Alternative mechanisms involving directly reactive granulocytes or cytophilic antibodies were rarely operative in the migration tests.


Br. J. Cancer (1976) 34, 14

### MECHANISMS OF LEUCOCYTE MIGRATION INHIBITION BY BREAST TUMOUR CELL FRACTIONS

B. M. JONES*

*From the Department of Immunology, Tenovus Research Laboratories, Velindre Hospital, Whitchurch, Cardiff*

Received 27 November 1975 Accepted 24 March 1976

**Summary.—Leucocyte migration inhibition by autologous breast tumour cell fractions was mediated by a soluble factor synthesized and released by mononuclear leucocytes and active against migrating granulocytes. This mechanism is similar to that previously described in respect to cell-mediated sensitivity to microbial antigens. Alternative mechanisms involving directly reactive granulocytes or cytophilic antibodies were rarely operative in the migration tests.**

THE leucocyte migration test (LMT) of Søborg and Bendixen (1967) has been successfully employed in studies of tumour-directed immune responses in patients with breast cancer (Andersen *et al.*, 1970; Cochran *et al.*, 1974; Jones and Turnbull, 1974, 1975; McCoy *et al.*, 1974); malignant melanoma (Cochran, Jehn and Gothoskar, 1972; McCoy *et al.*, 1975); hypernephroma (Kjaer, 1974); and various other tumours (Wolberg, 1971; Segall *et al.*, 1972). Significant discrimination between patients and controls was achieved, and the possibility exists that this simple and reproducible assay might be of considerable value in the clinical assessment of patients with cancer.

Mechanisms involved in LMT have mainly been studied in respect of sensitization by bacterial, fungal and viral antigens, and it was thought that in most cases inhibition was mediated by a soluble factor (leucocyte inhibitory factor, LIF) released by sensitized lymphocytes in the presence of specific antigen (Søborg, 1969; Clausen, 1973; Rocklin, 1974; Hoffman *et al.*, 1975). Alternative mechanisms thought to be operative under certain circumstances included reactions between antigens and cytophilic antibodies on the surface of leucocytes (Packalen and Wasserman, 1971; Rocklin, 1974) and the direct action of antigen on sensitized granulocytes (Senyk and Hadley, 1973). The present study provides evidence that leucocyte migration inhibition by particulate breast tumour cell fractions is mediated by mononuclear cells and is probably a manifestation of cellular immunity.

### MATERIALS AND METHODS

*Patients.*—The patients studied form part of an on-going therapeutic survey comparing simple mastectomy for Stage I or Stage II breast cancer with simple mastectomy plus radical radiotherapy. The control group consisted of healthy hospital workers, hospital in-patients with malignancies of organs other than breast and patients with benign breast disease.

*Preparation of tumour cell fractions.*—The preparation of particulate extracts from malignant breast tumours has been fully described elsewhere (Jones and Turnbull, 1975). Briefly, tissue obtained at operation was minced and disrupted using a dounce-type hand homogenizer and the homogenate centrifuged at 1000 *g* to remove nuclei. The supernatant (extract) was also further centrifuged at 3000 *g* and the sediment (3000 *g* fraction) retained.

* Current address: K.R.U.F. Institute of Renal Disease, Royal Infirmary, Newport Road, Cardiff.

*Separation of leucocytes*.—25-ml samples of blood were taken into 20 units/ml preservative-free heparin. 6-ml aliquots were layered on to 4 ml Ficoll-Triosil (FT), sp. gr. 1·07–1·08, and erythrocytes allowed to sediment for 60 min at room temperature. The upper layer of leucocyte-rich plasma was centrifuged at 350 *g* for 5 min and the cell pellet washed 3 times in tissue culture medium TC 199 supplemented with 13 mM $NaHCO_3$, 20 mM HEPES buffer, 200 $\mu$g/ml L-glutamine, 300 i.u./ml penicillin and 300 $\mu$g/ml streptomycin.

To separate mononuclear leucocytes and granulocytes, leucocyte-rich plasmas were diluted with an equal volume of TC 199, layered on to further aliquots of FT and centrifuged at 400 *g* for 40 min. The white band of cells at the plasma–FT interface (mononuclears) and the cell pellet (granulocytes) were collected and washed 3 times in TC 199.

*Cell migration tests*.—Migration inhibition tests were performed using a semi-micro method developed specifically to utilize minimal quantities of tumour cell fractions and which was previously shown to give a high degree of reproducibility (Jones and Turnbull, 1975). Tumour extracts at 100 and 200 $\mu$g/ml in TC 199 + 10% foetal calf serum (FCS), tumour 3000 *g* fraction at 100 and 200 $\mu$g/ml, and control medium TC 199 + 10% FCS without antigen were added to consecutive wells of the Sterilin migration chamber. Three heat-sealed capillaries, each containing $6 \times 10^5$ cells, were carefully positioned within each well, coverslips added and migration allowed to proceed for 18 h at 37 °C. Migration areas were measured by projection microscopy and planimetry and migration indices (MI) for each dilution of antigen calculated as follows:

$$MI = \frac{\text{Mean migration area in tumour cell fraction}}{\text{Mean migration area in control medium}}$$

In a small number of cases, the effect of puromycin on leucocyte migration inhibition was examined, when protein synthesis inhibitor was included in the migration medium at a concentration of 10 $\mu$g/ml.

*Indirect assay for LIF*.—Mononuclear leucocytes from control subjects or from patients with breast cancer, $2 \times 10^6$/ml, were incubated for 3 days at 37 °C in the presence of 200 $\mu$g/ml tumour extract or 3000 *g* fraction, or in control medium TC 199 + 10% FCS, the culture volume being 0·8 ml. Culture supernatants were obtained by centrifugation at 1000 *g* for 10 min, and granulocytes from control subjects without breast cancer were used as migrating indicator cells for LIF produced during culture.

*Cytophilic antibodies*.—The presence of humoral factors able to passively sensitize control leucocytes was examined using the method of Amos *et al.* (1967). Plasmas were obtained in the course of isolating leucocytes from whole blood; 20 samples came from breast cancer patients who had given positive results in direct LMTs against autologous tumour fractions, while 6 were from control subjects. Thrice washed leucocytes or separated granulocytes were incubated for 1 h at room temperature in 4 ml TC 199 + 10% plasma. Cells were recovered by centrifugation, carefully washed 3 times in TC 199 and dispensed into capillaries for migration tests against tumour extracts and 3000 *g* fractions.

### RESULTS

### *Separated cell populations*

Microscopic examination of Jenner–Giemsa-stained smears revealed that mononuclear cell preparations contained 87–95 (mean 89) % lymphocytes, 3–12 (mean 10) % monocytes and 0–3 (mean 1) % granulocytes. Granulocyte preparations contained 0–5 (mean 2·5) % contaminating lymphocytes.

### *Control MI values*

In migration studies employing leucocytes from 58 control subjects and fractions from over 100 tumours, 2226 MI values were normally distributed about a mean of 0·975, standard deviation 0·085. The 95% confidence limits were therefore 0·805–1·145 and values $\leqslant$0·80 and $\geqslant$1·15 were considered to indicate migration inhibition and enhancement respectively.

### *Indirect assay for LIF*

Culture supernatants obtained when separated mononuclear cells from 10

control subjects were incubated in the presence of cell fractions from 24 breast tumours failed to inhibit the migration of control granulocytes (mean MI 0·97, range 0·82–1·13). Similar supernatants obtained by incubating mononuclear cells from breast cancer patients with autologous tumour extracts were inhibitory in 4/22 (18%) cases, with autologous 3000 *g* fractions giving 6/24 (25%) positive tests. In all, 10/24 (42%) patients responded to extract and/or 3000 *g* fraction by the indirect assay ($P < 0{\cdot}01$, $\chi^2$ with Yates' correction). When the same patients were examined by the direct LMT, 6/22 (27%) responded to autologous extract, 7/24 (29%) to autologous 3000 *g* fraction and 11/24 (46%) to at least one of the preparations. Agreement between the two methods occurred in 41/46 (89%) tests ($P < 0{\cdot}0001$, $\chi^2$ test; Table I).

TABLE I.—*Comparison of Direct (Leucocyte Migration Inhibition by Autologous Breast Tumour Fractions included in the Migration Medium) and Indirect (Granulocyte Migration Inhibition by Factors produced in Mononuclear Cell Cultures with Autologous Tumour Fractions) Assays for LIF. Agreement between the Two Methods Occurred in* 41/46 (89%) *Tests* ($P < 0{\cdot}0001$).

| LIF assay | a<br>+ to extract | b<br>+ to 3000 *g* fraction | + to a and /or b |
|---|---|---|---|
| Direct | 6/22 (27%) | 7/24 (29%) | 11/24 (46%) |
| Indirect | 4/22 (18%) | 6/24 (25%) | 10/24 (42%) |

### *Addition of puromycin to the leucocyte migration system*

Migration areas in medium containing 10 μg/ml puromycin were reduced on average by 40% compared with migration areas in medium without added protein synthesis inhibitor. Control MIs (5 subjects tested against fractions from 10 tumours) were close to unity whether or not this agent was included in the migration medium, although control MIs were higher when puromycin was included

TABLE II.—*Effect of Puromycin on Leucocyte Migration Inhibition. In All but One Case, the Response to Autologous Breast Tumour Cell Fractions was Abolished by Puromycin Included in the Migration Medium at* 10 *μg/ml* ($P < 0{\cdot}05$).

| Puromycin | a<br>+ to extract | b<br>+ to 3000 *g* fraction | + to a and/or b |
|---|---|---|---|
| Nil | 3/10 (30%) | 6/10 (60%) | 7/10 (70%) |
| 10 μg/ml | 1/10 (10%) | 1/10 (10%) | 1/10 (10%) |

(mean 1·04, range 0·91–1·29) than when it was omitted (mean 0·93, range 0·82–1·09). As shown in Table II, 7/10 breast cancer patients gave leucocyte migration inhibition by autologous extract and/or 3000 *g* fraction in the absence of puromycin, while only 1/10 patients remained reactive in the presence of this agent ($P < 0{\cdot}05$, $\chi^2$ with Yates' correction).

### *Comparison of leucocyte and granulocyte migration inhibition*

Leucocytes and separated granulocytes from 5 control subjects were used as migrating cells in tests against extracts and 3000 *g* fractions from 10 tumours. The mean MI for leucocytes was 0·98, range 0·84–1·13, while for granulocytes the mean was 0·95, range 0·84–1·16. Results shown in Table III indicated that although granulocytes from breast cancer patients were contaminated by up to 5% lymphocytes, migration was not inhibited in any of the tests against autologous breast tumour cell fractions. This was in contrast to the finding that leucocytes from the same patients were inhibited by autologous extract and/or 3000 *g* fraction in 5/10 cases ($P < 0{\cdot}05$, $\chi^2$ with Yates' correction). In view of the possibility that granulocyte response to LIF produced by contaminating lymphocytes, or the direct response of sensitized granulocytes to antigen, might be impaired due to additional cell separation procedures, isolated mononuclears and granulocytes were admixed to contain

TABLE III.—*Comparison of Leucocyte and Granulocyte Migration Inhibition. Granulocytes Containing 5% Contaminating Mononuclear Cells were Non-responsive in Migration Tests* ($P < 0 \cdot 05$), *while the Addition of Approximately* 30% *Mononuclear Cells Restored the Ability to Respond Directly to Tumour Cell Fractions*

| Cells | a + to extract | b + to 3000 *g* fraction | + to a and/or b |
|---|---|---|---|
| Leucocytes | 1/10 (10%) | 4/10 (40%) | 5/10 (50%) |
| Granulocytes | 0/10 | 0/10 | 0/10 |
| Leucocytes | 1/5 (20%) | 2/5 (40%) | 2/5 (40%) |
| Granulocytes, 70% + mononuclears, 30% | 2/5 (40%) | 2/5 (40%) | 2/5 (40%) |

TABLE IV.—*Failure of Plasmas from LMT⁺ Breast Cancer Patients to Passively Sensitize Control Leucocytes or Granulocytes*

| Plasma-treated cells | a + to extract | b + to 3000 *g* fraction | + to a and/or b |
|---|---|---|---|
| Leucocytes | 1/20 (5%) | 3/20 (15%) | 4/20 (20%) |
| Granulocytes | 0/20 (2/20 enhanced) | 0/20 (1/20 enhanced) | 0/20 (2/20 enhanced) |

approximately 30% lymphocytes. The migration of these cells, and of leucocytes from the same patients, were inhibited in 2/5 cases (Table III).

*Cytophilic antibodies*

When control leucocytes or granulocytes were incubated with plasmas from healthy individuals before performing migration tests against breast tumour extracts and 3000 *g* fractions, MI values were within the normal range in all cases (0·83–1·01, mean 0·91, for leucocytes; 0·89–1·09, mean 0·99, for granulocytes). The migration of control leucocytes was inhibited after incubation with plasmas from only 4/20 LMT$^+$ breast cancer patients, and although 2/20 plasmas caused enhanced migration of granulocytes, inhibition of these cells was not observed (Table IV).

DISCUSSION

Leucocyte migration inhibition by breast tumour cell fractions was apparently not mediated by directly reactive granulocytes, nor in the majority of cases were cytophilic antibodies involved in the *in vitro* response. Addition of puromycin to LMTs suppressed inhibition, suggesting that active protein synthesis occurred in the course of the response to antigen. Mononuclear leucocytes, the majority of which were lymphocytes, from many of the patients studied, released a soluble factor into the culture medium when incubated with autologous tumour fractions, and this factor was able to inhibit the migration of granulocytes from control subjects without breast cancer. Thus the primary mechanism of leucocyte migration inhibition by insoluble tumour cell fractions was thought to be similar to that previously described in studies of delayed hypersensitivity to soluble microbial antigens.

While lymphokine production is thought to be a manifestation of the cellular immune system, production of macrophage inhibitory factor (Rocklin *et al.*, 1974), chemotactic factor (Altman and Mackler, 1974) and interferon (Epstein, Kreth and Herzenberg, 1974) by B- as well as T-lymphocytes has recently been reported. Fimmel (1975) showed that E rosette-forming cells were required for the production of LIF in response to *E. coli* somatic antigen, PPD and influenza virus antigen, but further studies are clearly required to determine the lymphocyte subpopulation active in leucocyte migration inhibition by particulate tumour-derived antigens.

We have shown that granulocyte preparations containing as many as 5% lymphocytes were not inhibited by autologous breast tumour fractions, while the addition of approximately 30% lymphocytes restored reactivity. Fimmel (1975) obtained inhibition when granulocyte preparations containing less than 2% lymphocytes were allowed to migrate in the presence of specific microbial antigens, but further passages through Ficoll-Hypaque columns reduced the number of contaminating lymphocytes to less than 0·1% and abrogated migration inhibition. Thus it appeared that the LMT response to tumour-derived antigens requires higher numbers of lymphocytes in the migrating cell population than were needed for the response to microbial antigens. This possibly reflects the inherent weakness of the tumour-directed response and in particular suggests that a relatively low proportion of peripherally circulating lymphocytes are committed to anti-tumour activities. In this respect it is interesting to note that Ellis *et al.* (1975) observed a higher proportion of $LMT^+$ responses to breast tumour antigens using cells from the tumour-draining lymph node rather than peripheral blood leucocytes, presumably due to localization of tumour-reactive lymphocytes in the former population.

Although the tumour-directed LMT is able to discriminate successfully between patient and control groups on a statistical basis, it has not been possible to demonstrate sufficiently high numbers of positive results in patients with early breast cancer for the technique to be developed as a diagnostic procedure. Various modifications of the test itself have failed to overcome this problem, though it is possible that refinement of antigen preparation procedures might yield further progress. Early studies employed tumour homogenates partially clarified by low speed centrifugation (extracts, Andersen *et al.*, 1970) and we have shown that the additional use of a 3000 *g* fraction rich in large membranous cell fragments increases the rate of positive results (Jones and Turnbull, 1975). McCoy *et al.* (1974) have improved still further on our rate of approximately 50% patients positive at 7 days after simple mastectomy: using 3M KCl extracts of breast tumours, significant leucocyte migration inhibition was obtained in 20/26 (77%) pre- or immediately post-surgery breast cancer patients. Possibly the higher rate of positives was due to the fact that soluble antigens could be used at higher concentrations without causing non-specific inhibition of control leucocyte migration.

It was previously shown that serial postoperative measurements of tumour-directed leucocyte migration inhibition in breast cancer patients may be of relevance to the understanding of the complex host–tumour interrelationship and may provide information of clinical significance (Jones and Turnbull, 1975; Jones *et al.*, 1976). This paper has tried to confirm that the *in vitro* response to particulate tumour-derived antigenic material is mediated by cellular immune mechanisms and thus permit interpretation of the clinical correlations observed in breast cancer patients.

This project was financed by a grant from the Tenovus (Cardiff) organization. I should like to thank Mr A. R. Turnbull and Mr D. T. L. Turner of the Surgical Division, Royal South Hants Hospital, Southampton, for supplying blood and tissue samples, and Mrs M. Evans for efficient technical assistance.

### REFERENCES

Altman, L. C. & Mackler, B. G. (1974) Chemotactic Lymphokine Production by Human Thymus-derived (T) and Bone Marrow-derived (B) Lymphocytes. *Fedn Proc.*, **33**, 745.

Amos, H. E., Gurner, B. W., Olds, R. J. & Coombs, R. R. A. (1967) Passive Sensitization of Tissue Cells. II. Ability of Cytophilic Antibody to Render the Migration of Guinea-pig Peritoneal Exudate Cells Inhibitable by Antigen. *Int. Archs Allergy*, **32**, 496.

ANDERSEN, V., BJERRUM, O., BENDIXEN, G., SCHIØDT, T. & DISSING, I. (1970) Effect of Autologous Mammary Tumour Extracts on Human Leukocyte Migration Inhibition *in Vitro*. *Int. J. Cancer*, **5**, 357.

CLAUSEN, J. E. (1973) Migration Inhibitory Effect of Cell-free Supernatants from Tuberculin-stimulated Cultures of Human Mononuclear Leukocytes Demonstrated by Two-step MIF Agarose Assay. *J. Immun.*, **110**, 546.

COCHRAN, A. J., GRANT, R. M., SPILG, W. G., MACKIE, R. M., ROSS, C. E., HOYLE, D. E. & RUSSELL, J. M. (1974) Sensitization to Tumour-associated Antigens in Human Breast Carcinoma. *Int. J. Cancer*, **14**, 19.

COCHRAN, A. J., JEHN, U. W. & GOTHOSKAR, B. P. (1972) Cell-mediated Immunity in Malignant Melanoma. *Lancet*, i, 1340.

ELLIS, R. J., WERNICK, G., ZABRISKIE, J. B. & GOLDMAN, L. I. (1975) Immunological Competence of Regional Lymph Nodes in Patients with Breast Cancer. *Cancer, N.Y.*, **35**, 655.

EPSTEIN, L. B., KRETH, W. & HERZENBERG, L. A. (1974) Fluorescence-activated Cell Sorting of Human T and B Lymphocytes. II. Identification of the Cell Type Responsible for Interferon Production and Cell Proliferation in Response to Mitogens. *Cell. Immun.*, **12**, 407.

FIMMEL, P. J. (1975) Studies on Leukocyte Migration Inhibition: the Role of E Rosette-forming Cells in Specific Antigen-induced Inhibition of Migration. *J. Immun.*, **115**, 135.

HOFFMAN, P. M., SPITLER, L. E., HSU, M. & FUDENBERG, H. H. (1975) Leukocyte Migration Inhibition in Agarose. *Cell. Immun.*, **18**, 21.

JONES, B. M., CONNOLLY, C. E., ISAACSON, P., TURNER, D. T. L. & TURNBULL, A. R. (1976) Tumour-directed Leucocyte Migration Inhibition in Operable Breast Cancer: Additional Clinical Correlations. *Br. J. Cancer*, **34**, 94.

JONES, B. M. & TURNBULL, A. R. (1974) *In Vitro* Cellular Immunity in Mammary Carcinoma. *Br. J. Cancer*, **29**, 337.

JONES, B. M. & TURNBULL, A. R. (1975) Horizontal Studies of Cell-mediated Immune Reactions to Autologous Tumour Antigens in Patients with Operable Mammary Carcinoma. *Br. J. Cancer*, **32**, 339.

KJAER, M. (1974) *In Vitro* Demonstration of Cellular Hypersensitivity to Tumour Antigens by means of the Leukocyte Migration Technique in Patients with Renal Carcinoma. *Eur. J. Cancer*, **10**, 523.

MCCOY, J. L., JEROME, L. F., DEAN, J. H., CANNON, G. B., ALFORD, T. C., DOERING, T. & HERBERMAN, R. B. (1974) Inhibition of Leukocyte Migration by Tumour-associated Antigens in Soluble Extracts of Human Breast Carcinoma. *J. natn. Cancer Inst.*, **53**, 11.

MCCOY, J. L., JEROME, L. F., DEAN, J. H., PERLIN, E., OLDHAM, R. K., CHAR, D. H., COHEN, M. H., FELIX, E. L. & HERBERMAN, R. B. (1975) Inhibition of Leukocyte Migration by Tumour-associated Antigens in Soluble Extracts of Human Malignant Melanoma. *J. natn. Cancer Inst.*, **55**, 19.

PACKALEN, T. & WASSERMAN, J. (1971) Inhibition of Migration of Normal Guinea-pig Blood Leukocytes by Homologous Immune $\gamma_2$-globulin in the Presence of Specific Antigen. *Int. Archs Allergy Appl. Immun.*, **41**, 790.

ROCKLIN, R. E. (1974) Products of Activated Lymphocytes: Leukocyte Inhibitory Factor (LIF) Distinct from Migration Inhibitory Factor (MIF). *J. Immun.*, **112**, 1461.

ROCKLIN, R. E., MACDERMOTT, R. P., CHESS, L., SCHLOSSMAN, S. F. & DAVID, J. R. (1974) Studies on Mediator Production by Highly Purified Human T and B Lymphocytes. *J. exp. Med.*, **140**, 1303.

SEGALL, A., WEILER, O., GENIN, J., LACOUR, J. & LACOUR, F. (1972) *In Vitro* Study of Cellular Immunity against Autochthonous Human Cancer. *Int. J. Cancer*, **9**, 417.

SENYK, G. & HADLEY, W. K. (1973) *In Vitro* Correlates of Delayed Hypersensitivity in Man: Ambiguity of Polymorphonuclear Neutrophils as Indicator Cells in Leukocyte Migration Tests. *Infect. Immun.*, **8**, 370.

SØBORG, M. (1969) The Interaction of Lymphocytes and Granulocytes in the Migration–Inhibition Reaction. *Acta med. scand.*, **185**, 221.

SØBORG, M. & BENDIXEN, G. (1967) Human Lymphocyte Migration as a Parameter of Hypersensitivity. *Acta med. scand.*, **181**, 247.

WOLBERG, W. H. (1971) Inhibition of Migration of Human Autogenous and Allogeneic Leukocytes by Extracts of Patients' Cancers. *Cancer Res.*, **31**, 798.